# Zero-echo time MRI: an alternative method for the diagnosis of urinary stones in children

**DOI:** 10.1007/s00330-024-10950-x

**Published:** 2024-07-12

**Authors:** H. Nursun Ozcan, Gozde Ozer, Hasan Serkan Dogan, Jale Karakaya, Berna Oguz, Serdar Tekgul, Mithat Haliloglu

**Affiliations:** 1 Division of Pediatric Radiology, Department of Radiology, Hacettepe University School of Medicine, Ankara, Turkey; 2 Division of Pediatric Urology, Department of Urology, Hacettepe University School of Medicine, Ankara, Turkey; 3 Department of Biostatistics, Hacettepe University School of Medicine, Ankara, Turkey

**Keywords:** Pediatric, Renal stone, CT, MRI

## Abstract

**Objectives:**

To evaluate the potential of zero-echo time-magnetic resonance imaging (ZTE-MRI) in the assessment of urolithiasis and compare ZTE-MRI with computed tomography (CT) in pediatric patients.

**Materials and methods:**

This was a single-center, prospective cross-sectional study conducted between April 2023 and December 2023. 23 patients (12 girls, 11 boys; mean age: 12.3, range 1–18) with urinary tract stones detected on non-enhanced abdominal CT were enrolled. The images were evaluated independently by two radiologists for the presence, and number of stones in the kidneys, ureters, and bladder. In the second session, two radiologists evaluated whether urinary tract stones could be detected by MRI compared to CT, and the maximum diameter of the stones was measured. The CT and MRI results were compared with the Wilcoxon test. The agreement between the results of the observers was examined using Spearman’s rho correlation coefficient and the intraclass correlation coefficient.

**Results:**

A total of 58 urinary tract stones were detected by CT and 39 of these were detected by MRI. Most of the stones that MRI could not detect were < 5 mm and the detection sensitivity of MRI increased in correlation with stone size (*p* < 0.001). There was poor intermodality agreement for stones < 5 mm, substantial agreement for stones 5–10 mm, and almost perfect agreement for stones > 10 mm. Interobserver agreement for stone detection on MRI was almost perfect for stones > 10 mm and 5–10 mm and was substantial for stones < 5 mm.

**Conclusion:**

ZTE-MRI is a promising modality for detecting urinary stones without radiation exposure in children.

**Clinical relevance statement:**

Zero-echo time-magnetic resonance imaging is a potential method for identifying urinary stones in children and other populations who are particularly sensitive to radiation.

**Key Points:**

- *Urinary system stone disease in children is increasing and imaging is needed for managing urolithiasis.*
- *Zero-echo time-magnetic resonance imaging (ZTE-MRI) had an accuracy of 81.8% and 93.7% for stones larger than 5* *mm and 10* *mm, respectively*.
- *ZTE-MRI is a potential non-irradiating method for the diagnosis and management of urolithiasis.*

## Introduction

Urolithiasis is a common problem affecting people in all age groups. The prevalence of urinary stone disease in children has significantly increased during the past 10 or so years [1]. Typical signs and symptoms of urolithiasis include flank or abdominal pain and macroscopic or microscopic hematuria; fever, nausea, and vomiting may also occur. Urolithiasis can initially be misdiagnosed due to unclear symptoms, especially in newborns and infants. Numerous pediatric patients with kidney stones stay asymptomatic and are diagnosed incidentally on imaging examinations.

Ultrasonography (US) is a non-ionizing, widely available, and effective method that is recommended as the first diagnostic tool for urolithiasis in children. Conventional radiography, which includes the kidney, ureter, and bladder, has low sensitivity and specificity when used alone, and furthermore, not all stones are radiopaque therefore, in combination with US is recommended for selected pediatric patients [2–4].

The gold standard examination for the diagnosis of urolithiasis in adults is non-enhanced computed tomography (CT) [5]. It can be used to define the definitive location, size, and shape of nearly all stones, including those found in the ureters. The main disadvantage of CT is ionizing radiation, especially in children. Non-enhanced CT is the gold standard imaging modality regarding the diagnosis of urolithiasis since it has high sensitivity (97–100%) and specificity (96–100%) [2, 3].

Zero-echo time (ZTE) is a novel magnetic resonance imaging (MRI) technique that performs images similar to those obtained with radiography or CT with the shortest T2 values. ZTE is currently primarily used in musculoskeletal imaging protocols [6]. Recently, lung ZTE-MRI may provide additional information in detecting and differentiating lung lesions [7]. The primary aim of this study was to evaluate the potential of ZTE-MRI in the assessment of urolithiasis and compare ZTE-MRI with CT in pediatric patients.

## Materials and methods

### Patients

**Fig. 1:**
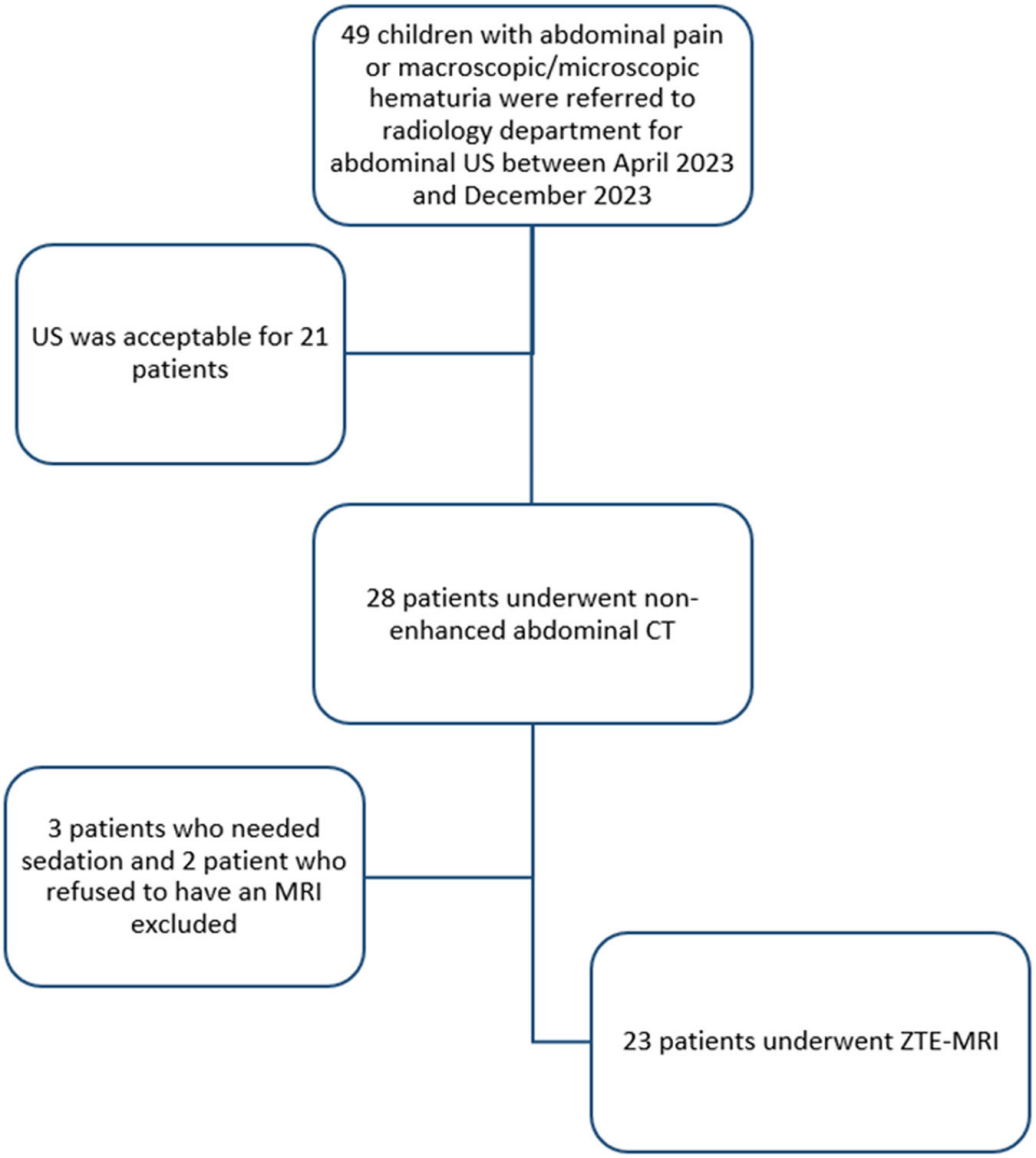
Flowchart shows the selection of the study sample

A prospective study was conducted after approval by our institutional ethics committee. Between April 2023 and December 2023, 49 patients who presented with abdominal pain and hematuria were referred for abdominal US examination with suspicion of urolithiasis. US examination was sufficient in 21 patients. 28 of these patients were referred from the Pediatric Urology Department to the Radiology Department for further evaluation, where a non-enhanced CT scan was performed. Three patients who needed sedation and two patients who refused to have an MRI were excluded. A total of 23 patients (12 girls, 11 boys; mean age: 12.3, range 1–18) underwent an MRI of the abdomen within 24 h of the CT scan (Fig. 1). Informed consent was obtained from all relatives of the patients. Informed assent was obtained from all children who were eligible for the study (aged 8 years and older).

### CT protocol

Non-enhanced abdominal CTs were performed on a GE LightSpeed 16-slice CT Scanner (GE Healthcare). Standard non-enhanced abdominal CT was obtained with 2.5-mm contiguous sections on the axial plane, weight-based kilo voltage, low-dose tube current (10 mA), high-speed mode; and standard soft-tissue algorithm reconstruction. The reformatted slice thickness was 3 mm in the axial and coronal planes. The imaging volume was extended from the upper pole of both kidneys to the base of the urinary bladder.

### MRI protocol

MRI examinations were performed without contrast administration using a 3 T GE Signa Architect Scanner (GE Healthcare) with a 16-channel body coil. Images were obtained in axial and coronal planes with ZTE. Coronal T2 single-shot fast spin echo (SSFSE) sequence was obtained if there was pelvicalyceal dilatation. Sequence parameters are summarized in Table 1. The total acquisition time was about 10 min. Sedation was not performed on any of the patients. The 1-year-old patient underwent an MRI during sleep to minimize motion artifacts.

**Table 1:** MRI sequence parameters

Parameters	Axial ZTE	Coronal ZTE	Coronal T2 SSFSE
TR/TE (ms)	625.9/0	625.9/0	1200/30
Flip angle (degrees)	1	1	90
Field of view (mm)	300 × 300	380 × 380	420 × 420
Slice thickness (mm)	2	2	6
Acquisition time	4 m 14 s	4 m 14 s	33 s

*SSFSE* single-shot fast spin echo, *ZTE* zero-echo time

### Image analysis

All US evaluations were performed in the radiology department. CT and MRI examinations were independently evaluated on the PACS by two pediatric radiologists with 12 years (H.N.O.) and 2 years of experience (G.O.) in pediatric body imaging. Radiologists interpreted CTs and MRIs at 1-week intervals to reduce bias, first MRI then CT examinations evaluated. Images were assessed for the presence and number of stones in the kidneys, ureters, and bladder. The maximum diameter of the stone on the axial or coronal plane was measured, and stones were classified as 1–5 mm, 5–10 mm, and > 10 mm. Urinary tract dilatation was also recorded. Subsequently, the readers performed a second look session in consensus and compared each of the MRI examinations with CT examinations. In this second session, the two radiologists assessed whether the urinary tract stones could be detected with MRI when the CT scans served as a direct comparison. Also in this second session, the maximum diameter of the stones was measured for evaluating intermodality agreement.

### Statistical analysis

CT and MRI results were compared separately for observers using the Wilcoxon test. Median (min–max) values or count (percentage) are given as descriptive statistics. The agreement in the results of the observers was examined with Spearman’s rho correlation coefficient and Intra-class correlation coefficient (ICC). The agreement of the size measurements between the two modalities was examined with Pearson’s correlation coefficient and ICC. The interpretation of the ICC was made using Landis and Koch’s description [8]. Statistical analysis was performed with the IBM SPSS Statistics 23.0 statistics software package. The statistical significance level was determined as *p* < 0.05.

## Results

**Fig. 2:**
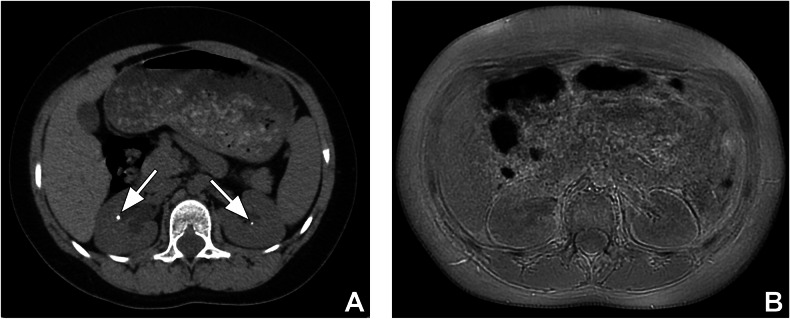
A 9-year-old boy with bilateral renal stones. **A** Axial non-enhanced CT image shows bilateral < 5 mm renal stones (arrows). **B** Failure to detect stones on axial ZTE-MR image

All CTs and MRIs were acceptable in terms of image quality. A total of 58 urinary tract stones were detected by CT and 39 of these were detected by MRI. Most of the stones that MRI could not detect were < 5 mm and the detection sensitivity of MRI increased in correlation with stone size (*p* < 0.001) (Fig. 2). The sensitivity of ZTE-MRI and the intermodality agreement between CT and ZTE-MRI for stone detection are given in Table 2. There was poor intermodality agreement for stones < 5 mm, substantial agreement for stones 5–10 mm, and almost perfect agreement for stones > 10 mm.

**Table 2:** Sensitivity for ZTE-MRI and intermodality agreement for stone detection between ZTE-MRI and CT for urinary tract stones

Stone size	Number of detected stones on CT	Number of detected stones on MRI	Sensitivity for ZTE MRI (95% CI)	ICC (95% CI)	Spearman’s *r*
1–5 mm	20	6	30% (11.89–54.28)	−0.050 (−0.445–0.362)	0.017
5–10 mm	22	18	81.82% (59.72–94.81)	0.683 (0.385–0.852)	0.423
> 10 mm	16	15	93.75% (69.77–99.84)	0.966 (0.923–0.926)	0.861

*CI* confidence interval, *ICC* intraclass correlation coefficient, *ZTE* zero-echo time

**Fig. 3:**
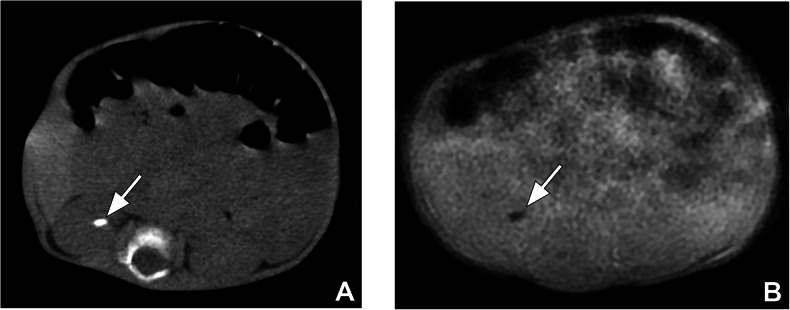
A 1-year-old girl with a right renal stone. **A** Axial non-enhanced CT and (**B**) ZTE-MR images show renal stone on the right side (arrows)

**Fig. 4:**
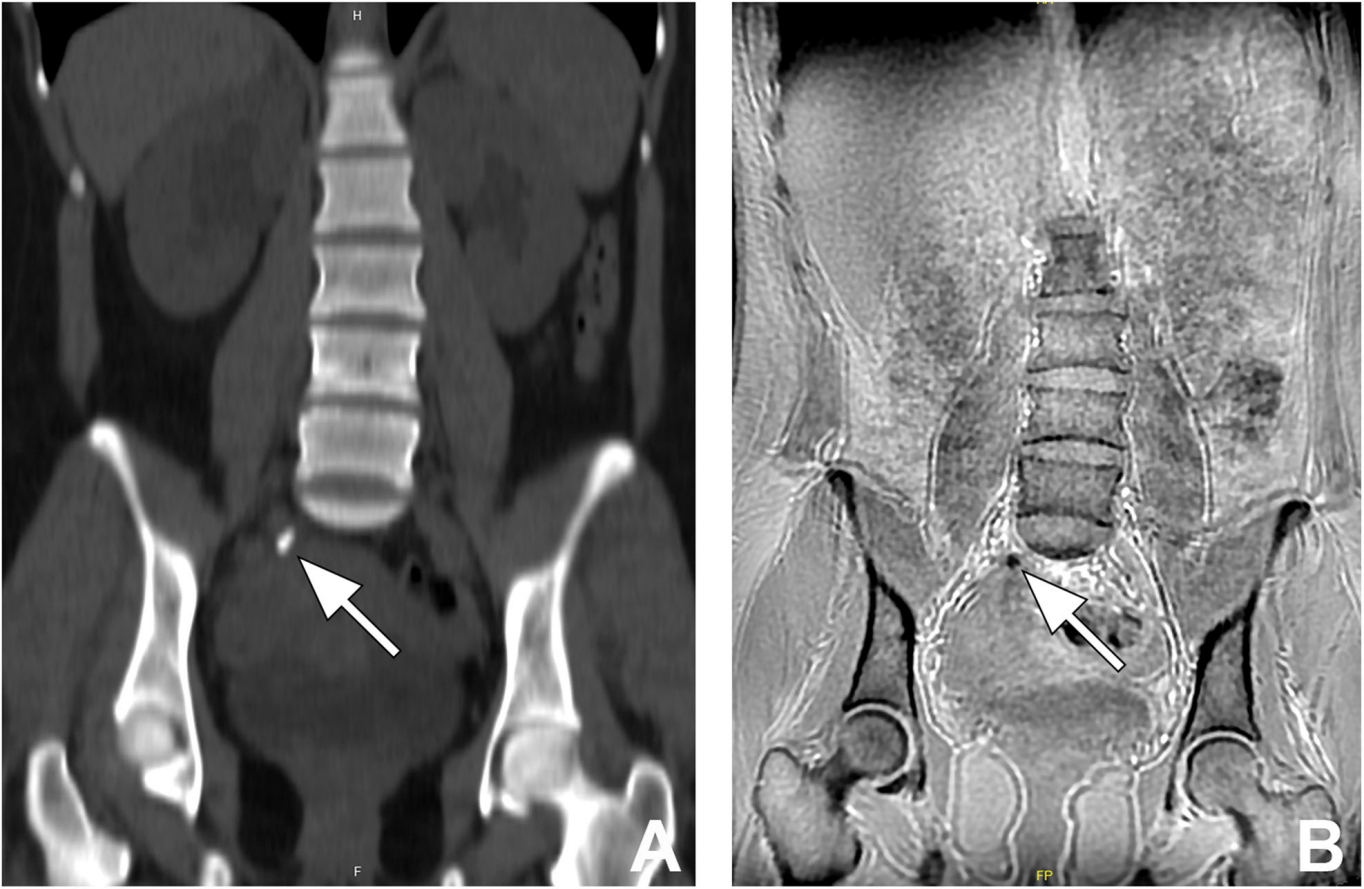
A 17-year-old girl with a right ureter stone. **A** Coronal non-enhanced CT image and (**B**) coronal ZTE-MR image at the same level show the stone (arrow) in the right ureter

In the right kidney, CT detected 12 stones < 5 mm and only one stone was detected by MRI (*p* = 0.031). CT detected 10 stones 5–10 mm in size and 6 stones > 10 mm; all of them were detected by MRI (Fig. 3). However, one stone measuring 5–10 mm was classified as > 10 mm. Thus, 9 stones 5–10 mm and 7 stones > 10 mm in size were detected by MRI (*p* = 0.317). In the right ureter, CT detected 5 stones (2 stones < 5 mm, 2 stones 5–10 mm, and 1 stone > 10 mm), and all of them were detected and classified as the right size group by MRI (Fig. 4). Urinary tract dilatation was detected in 8 patients on the right side.

**Fig. 5:**
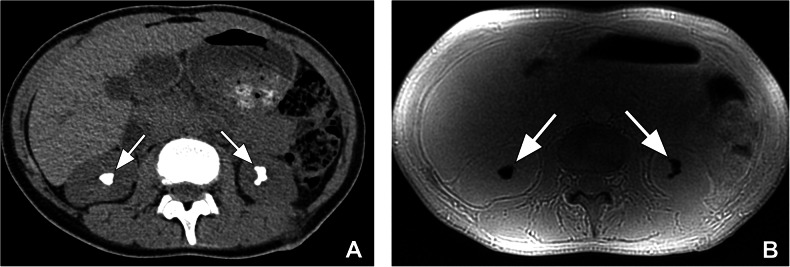
A 15-year-old girl with bilateral kidney stones. **A** Axial non-enhanced abdominal CT image shows lower pole stones in the right and left kidneys (arrows). **B** Axial ZTE-MR image at the same level demonstrates kidney stones (arrows)

In the left kidney, CT detected 5 stones < 5 mm, and only 1 stone was detected by MRI (*p* = 0.257). CT detected 6 stones 5–10 mm in size, and 4 stones were detected by MRI (*p* = 0.317). CT detected 5 stones > 10 mm, and all of them were detected by MRI (*p* = 1) (Fig. 5). In the left ureter, CT detected 6 stones (1 stone < 5 mm, 4 stones 5–10 mm, and 1 stone > 10 mm), and all of them were detected and classified as the right size group by MRI. Urinary tract dilatation was detected in 5 patients on the left side.

In the bladder, CT detected 3 stones > 10 mm and all were detected by MRI; however, one of them was classified as 5–10 mm.

The results of interobserver agreement for stone detection for CT and ZTE-MRI are summarized in Table 3. There was almost perfect agreement between readers in terms of stone detection on CT, regardless of stone size. Interobserver agreement for stone detection on MRI was almost perfect for stones > 10 mm and 5–10 mm and was substantial for stones < 5 mm.

**Table 3:** Interobserver agreement between ZTE-MRI and CT for stone detection

	Reader 1	Reader 2	ICC (95% CI)	Spearman’s *r*
**CT**				
< 5 mm	20	20	0.979 (0.950–0.991)	0.991
5–10 mm	22	21	0.948 (0.881–0.978)	0.980
> 10 mm	16	17	0.990 (0.976–0.996)	1
**ZTE MRI**				
< 5 mm	6	7	0.682 (0.384–0.852)	0.683
5–10 mm	18	16	0.928 (0.837–0.969)	0.835
> 10 mm	15	16	0.990 (0.976–0.996)	0.924

*CI* confidence interval, *ICC* intraclass correlation coefficient, *ZTE* zero-echo time

**Fig. 6:**
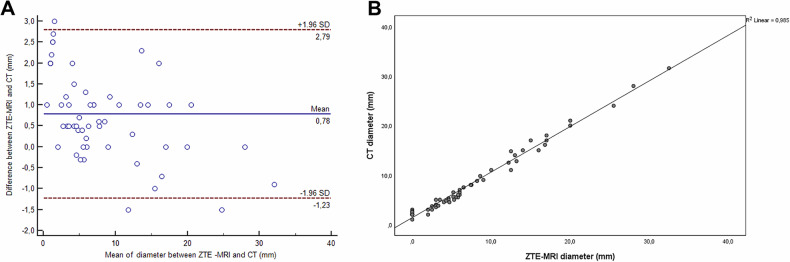
**A** Inter-modality correlation and (**B**) agreement for urinary tract stone measurements

In the evaluation made in the second session, 47 of 58 stones were detected by ZTE-MRI. The 58 stones had a mean diameter of 8.4 mm (min–max: 1–31.6 mm) on CT and 7.7 mm (min-max: 0–32.5 mm) ZTE-MRI. The inter-modality ICC value was 0.995 (95% confidence interval: 0.991, 0.997) for diameter. Scatter plots show a strong correlation (Pearson’s *r*: 0.993) between CT and ZTE-MRI (Fig. 6A). Bland-Altman plots (Fig. 6B) demonstrate the inter-modality agreements between CT and ZTE-MRI, with the means and limits of the agreement being 0.78 (−1.23, 2.79).

## Discussion

ZTE-MRI is a promising imaging technique for the assessment of urinary stones in children. The comparison of ZTE-MRI and CT in our study showed an overall diagnostic accuracy of MRI, especially for stones larger than 5 mm and 10 mm (81.8% and 93.7% sensitivity, respectively). Moreover, ZTE-MRI revealed almost perfect inter-modality and inter-observer agreement with CT for the diameter measurement of the urinary stones.

For pediatric patients, the US examination is the first diagnostic test that has the advantages of being readily available and radiation-free. Although the accuracy of the procedure depends on the experience of the physician, the sensitivity and specificity of kidney stone detection are 61–93% and 95–100%, respectively [2, 9]. The US detection rate is significantly higher for stones in the kidney than in the ureter [10].

Non-enhanced CT has the highest sensitivity (97–100%) and specificity (96–100%) for the detection of urinary stones [11]. However, one should be aware of the risks associated with exposure to ionizing radiation, especially in pediatric patients, such as the potential risk of developing a hematologic or solid malignancy [12]. This awareness has led to the introduction of ultra-low dose CT protocols that aim to reduce the radiation dose without compromising the quality of imaging. The recurrent nature of urinary stone disease in children should also be taken into consideration during the radiological evaluation of these patients.

MRI is a radiation-free technique that produces images with high tissue contrast and high resolution. Visualization of kidney stones relies on creating a strong contrast between a low signal stone and the surrounding structures, which was possible with few sequences [13]. This was mainly because most of the water molecules associated with these stones were too “solid” to provide a strong signal on conventional MRI pulse sequences, so MRI produces images of mobile water molecules. MRI is more time-consuming and expensive and requires sedation, especially in young children. Because of these facts mentioned above, conventional MRI techniques are not actively used for the assessment of urinary stones, and their use for urolithiasis is experimental and mainly limited to pregnancy periods [14].

The development of pulse sequences such as ZTE and Ultra Short Echo Time sequences are methods for recording proton signals in a short time before they fade. These methods have been shown to facilitate practical MRI-based imaging of bone, cartilage, lung parenchyma, tendons, and atherosclerotic plaques [15].

Besides being radiation-free, T2-weighted MRI sequences provide anatomic details better, which can be an advantage in patients who are candidates for surgical intervention. Especially for kidney stone cases, those will undergo endoscopic stone surgery (retrograde intrarenal surgery or percutaneous nephrolithotomy), MRI can be advantageous in terms of correct calyceal location of the stone, delineating the calyceal anatomy, infundibular length, width, infundibulopelvic angle, and neighboring organ relations those are important in decision making of type of intervention. The evaluation of these anatomical details is more difficult, and calculations are arbitrary by non-enhanced CT.

To the best of our knowledge, no study -even in adults- using ZTE-MRI for the detection of kidney stones has been performed to date. According to our results, of 58 stones detected on CT, 39 (67.2%) could be visualized on MRI. In addition, ZTE-MRI was able to detect and classify the correct size of all ureteral stones, which are more difficult to detect with the US method. The interobserver agreement in detecting stones with ZTE-MRI was almost perfect for stones > 10 mm and 5–10 mm and substantial for stones < 5 mm.

Non-enhanced CT can detect small-size (< 5 mm) ureteral stones better than other imaging methods. Although diagnosis is very important to clarify the symptoms of the patient, more than 90% of these cases do not need intervention and spontaneous passage is evident with a conservative approach. Non-enhanced CT and ZTE-MRI have similar diagnostic accuracy for kidney stones, especially for moderate to large sizes that most of the time require surgical intervention. The location and pelvicalyceal anatomy are important during decision-making for the surgical modality. Our MRI protocol (ZTE with T2-weighted sequence) may provide similar anatomical details as non-enhanced CT without ionizing radiation exposure. These results suggest that MRI has significant potential, particularly for medium to large kidney stones that are candidates for surgical intervention. Stones smaller than 5 mm may be of clinical significance and are usually presented on admission to the emergency department. A non-enhanced CT may be reserved for patients with suspected small stones. A new strategy for pediatric stone disease can be drawn as the US can be used for the first step evaluation or emergent cases, as the second step MRI can be used for moderate to larger size stones for the age groups who do not need sedation as a preparation imaging study for elective surgery and CT may be reserved for assessment of inconclusive cases with the US or in cases with suspicion of possible small stones.

Our study had some limitations. First, because of the study design and inclusion criteria, the patient group was limited. We included only 23 children with 58 urinary stones. Since we do not have a similar previous study, we did not have the opportunity to make a power analysis. Further studies with larger sample sizes evaluating the additional parameters may be conducted. Second, in our study, only two readers evaluated CTs and MRIs 1 week apart. Third, our study was performed with 3-T MRI, its applicability with 1.5 T was not investigated. However, 3-T MRI is known to give better results due to its fast transmit-receive switching, high gradient performance, precise radiofrequency waveform transmission, and shorter imaging time [6]. Finally, in our cohort, all patients had stones on CT, thus false positives and specificity of MRI were not assessed.

In conclusion, our results indicate that ZTE-MRI is a promising modality for detecting urinary stones without radiation exposure in children and highlights its potential benefits for kidney stones that will undergo surgical intervention.

## Abbreviations

CI: Confidence interval
CT: Computed tomography
ICC: Intraclass correlation coefficient
MRI: Magnetic resonance imaging
SSFSE: Single-shot fast spin echo
US: Ultrasonography
ZTE: Zero-echo time
